# pH- and Facet-Dependent Surface Chemistry of TiO_2_ in Aqueous Environment from First Principles

**DOI:** 10.1021/acsami.2c19273

**Published:** 2023-02-14

**Authors:** Farahnaz Maleki, Giovanni Di Liberto, Gianfranco Pacchioni

**Affiliations:** Dipartimento di Scienza dei Materiali, Università di Milano-Bicocca, via R. Cozzi 55, 20125 Milano, Italy

**Keywords:** DFT, pH dependence, ab initio molecular dynamics, solid/water interface, TiO_2_

## Abstract

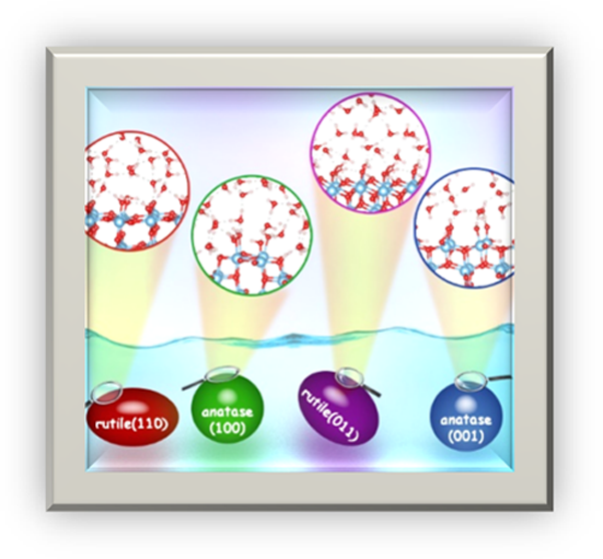

TiO_2_ is a relevant catalytic material, and its chemistry in aqueous environment is a challenging aspect to address. Also, the morphology of TiO_2_ particles at the nanoscale is often complex, spanning from faceted to spherical. In this work, we study the pH- and facet-dependent surface chemistry of TiO_2_/water interfaces by performing ab initio molecular dynamics simulations with the grand canonical formulation of species in solution. We first determined the acid–base equilibrium constants at the interface, which allows us to estimate the pH at the point of zero charge, an important experimental observable. Then, based on simulated equilibrium constants, we predict the amount of H^+^, OH^–^, and adsorbed H_2_O species present on the surfaces as a function of the pH, a relevant aspect for water splitting semi-reactions. We approximated the complex morphology of TiO_2_ particles by considering the rutile (110) and (011), and anatase (101), (001), and (100) surfaces.

## 1 Introduction

The description of the nature of the oxide/water interface is a relevant topic for photocatalysis, geochemistry, and materials science in general.^1^ Considering that most applications are done in aqueous environment, the fundamental understanding of how water interacts with the surface of a material is of fundamental importance and opens the way toward several applications. Several studies are devoted to the characterization of the nature of water on a catalytic material and in particular on oxide surfaces. Indeed, the local structure of water molecules can be very different from that of bulk water, and H_2_O can either adsorb molecularly, or it can dissociate on the surface sites.^2^ In addition, the interaction of a surface with water can stabilize the system or induce dissolution/degradation processes.

Once established how water interacts with a surface, an aspect of equal importance is the accurate description of acid–base equilibria since this has various implications ranging from chemical biology to geochemistry.^3^ For instance, in the field of catalysis, the measure of the acid–base equilibria at solid/water interfaces allows one to access very important information as the concentration of H^+^, OH^–^, and adsorbed H_2_O species at a surface as a function of the pH.^4^ At the same time, the estimate of equilibrium constants allows one to provide a measure of the proton transfer process, attracting attention from both experimental and theoretical chemists.^5,6^

A relevant experimental observable in this respect is the point of zero charge (PZC),^7^ corresponding to the pH at which the concentration of H^+^ on the surface equals that of OH^–^. This quantity can be obtained once the equilibrium constants at the solid/water interface are known.^8^ Given the intrinsic atomistic nature of problem, the quantum mechanical treatment of solid/water interface is needed. Interestingly, the prediction of the pH_PZC_ creates a direct link between the atomistic nature and macroscopic behavior of a system.

Several theoretical methodologies can be invoked to predict the equilibrium constants and pH_PZC_.^8−10^ One of these methodologies has been proposed by Pasquarello and co-workers and is based on the grand canonical formulation of species in solution, where the nature of the solid/water interfaces and equilibrium constants can be obtained through ab initio molecular dynamics simulations (AIMD).^8^ The state-of-the-art methodology to approximate the electronic energy is based on density functional theory (DFT).^11^

A widely applied and studied material is titanium dioxide mainly because of its manyfold applications areas, ranging from photocatalysis to paintings. TiO_2_ is mainly present in three different polymorphs, anatase, rutile, and brookite.^12^ The first two are of primary importance in catalysis, and their catalytic activity can be tailored by properly engineering the exposed surfaces.^13−15^ The latter, brookite, is usually less stable, but it was successfully interfaced with anatase finding an interesting photocatalytic activity.^16^ TiO_2_ is a challenging system given the possibility to grow different polymorphs and the tendency to interact with water.^17−19^ This can have various implications and important studies have been dedicated to understand the reactivity of water with different TiO_2_ surfaces.^20−22^ For instance, it has been observed that the dissociation of water is more likely to occur on the anatase (001) surface, rather than the more stable (101) one.^23^ At the same time, the rutile (110) surface mainly forms stable adducts of physisorbed water molecules.^24^

In this work, we investigate the pH-dependent surface chemistry of TiO_2_ nanoparticles in an aqueous environment. Given the complex morphology of TiO_2_ particles that can range from faceted to spherical shape,^18^ we adopt a simplified framework considering the most relevant surfaces. More specifically, we simulate stable surfaces of anatase and rutile that can reasonably approximate the thermodynamic behavior of a particle, and we consider less stable surfaces to account for reactive sites. We do not consider extended defects such as kinks and steps, although they can impact the surface chemistry. We consider the (110) and (011) surfaces of rutile, two relevant and exposed surfaces of real titania particles. The (110) surface is the most stable one and the most important in Wulff crystal,^25^ but also the less stable (011) one is considered to be relevant.^26^ Moving to anatase, we considered the anatase (001) surface that contributes to the Wulff nanoparticle shape together with the (101) one.^27^ For anatase (101), we use results from our previous work.^9^ Finally, we will account for the (100) anatase surface that can be exposed by particles under certain synthetic conditions.^28^

We will show that the different behavior of water with various TiO_2_ surfaces has important implications on the pH-dependent surface chemistry, and on the availability of charged species that in turn could have implications for the reactivity of nanoparticles. Rutile (110) and (011) surfaces exhibit significant changes in reactivity with water, being water dissociation more favorable on the (011) than on the (110), and also the acid–base equilibria are very different. Similarly, anatase (001) induces water dissociation more easily than the (101) surface. The consequence is that charged species such as H^+^ and OH^–^ are more likely to be present in a large pH window on the (001) than on the (101) facet. Finally, the pH-dependent surface chemistry of anatase (100) reminds that of anatase (101).

In summary, atomistic simulations of different TiO_2_ surfaces in aqueous environment indicate that water interactions range from molecular to dissociative adsorption, and the acid–base equilibria is largely affected by the nature of the different surfaces. This strongly impacts the amount of available species at the surface, with relevant implications in catalytic processes. This study also shows the importance of controlling the morphology and surface exposure of a catalyst’s oxide nanoparticle.

## 2 Computational Details

The calculations have been performed using the plane-wave-based VASP code.^29,30^ The Perdew–Burke–Ernzerhof (PBE) exchange-correlation functional was adopted.^31^ The projector augmented wave (PAW) approach has been used for the treatment of core electrons,^32^ and valence electrons have been described with plane waves expanded with a cutoff of 400 eV. Each self-consistent field electronic energy cycle was considered converged by imposing a threshold of 10^–5^ eV. Dispersion forces have been considered according to the Grimme’s D3 parametrization scheme.^33^ Since PBE tends to underestimate the semiconducting behavior of TiO_2_, we adopted the PBE + *U* approach with a working Hubbard *U* term for Ti (3*d*) states equal to 3.3 eV.^34^*U* is usually chosen to improve the estimate of some quantity, such as the electronic structure. However, the choice of the *U* parameter can affect other quantities, and therefore the approach is not free from issues.^35^ At the same time, PBE also underestimates the highest occupied molecular orbital–lowest unoccupied molecular orbital (HOMO–LUMO) gap of water, which could affect the accuracy if one is interested in reproducing its insulating behavior.^36,37^ Also, PBE is not exceptional in OH binding.^38−40^ In this case, one should invoke hybrid functionals.^11,41^ Therefore, the choice of the functional is not trivial since one should adopt an approach sufficiently reliable for both the material and water, which does not result in too demanding calculations. Nonhybrid functionals, including PBE, were shown sufficiently reliable in providing estimates of the pH dependence of the surface chemistry of materials, and thus the choice of the DFT functional can be considered acceptable in this respect.^8,9^ Trajectories were propagated for 5 ps after two 2 ps of equilibration. The temperature has been set to 350 K, which guarantees a frank diffusive motion of liquid water,^8^ and controlled by the Nosé–Hoover thermostat.^42,43^

We fully optimized the bulk structure of rutile and anatase starting from the available experimental values using a tight sampling of reciprocal space.^12^ We then designed surface models and optimized the atomic coordinates. We considered the most relevant rutile low-index surfaces, (110) and (011), which are relevant in rutile nanoparticles.^25^ Moving to anatase, we simulated the reactive (001) surface,^27,44^ and the (100) surface that gained attention over the past few years because of its relative stability and high catalytic activity over specific reactions.^45^ The most stable anatase surface is the (101) one.^46^ Results on this system have been taken from our previous work for comparison.^9^ We report in Table 1 the structural parameters of the optimized clean surfaces. The quantities compare well with the experimental ones.^45,47−49^ Thickness of the surface model is an important parameter to account for to provide reliable estimates, and it has been discussed in the past.^50^ In general, one should adopt a sufficiently thick model to provide reasonably converged surface properties, as the surface energy. For instance, in the case of rutile (110), previous studies showed that at least a five-layer-thick model is necessary, the same adopted here.^51−53^

**Table 1:** Lattice Vectors, Thickness, and Surface Energy of Rutile (110), Rutile (011), Anatase (001), and Anatase (100) Models^a^,^54^

	*a*/Å	*b*/Å	*d*/nm	*E*_s_/J m^–2^
rutile (110)	2.999 (2.958)	6.552 (6.497)	1.6	1.66
rutile (011)	4.633 (4.593)	5.519 (5.463)	1.3	1.71
anatase (001)	3.852 (3.785)	3.852 (3.785)	1.4	0.93
anatase (100)	3.852 (3.785)	9.538 (9.514)	1.2	1.22

a Experimental numbers are reported in parentheses.

Finally, we generated supercells and interfaced them with water. A water box with a thickness of about 2 nm was created and was filled with a number of water molecules sufficient to guarantee a density of about 1 g·cm^–3^. To create the interface, one needs to find a compromise between computational effort and size of the simulation cells to maximize the degrees of freedom, including the number of possible adsorption sites. Based on previous works,^8,55^ we generated the following models. More specifically, a 3 × 1 rutile (110) supercell was interfaced, including 36 water molecules, resulting in 198 atoms in the simulation cell. A 2 × 1 rutile (011)/water model was created, made by 180 atoms in the simulation box, of which 36 water molecules. A 2 × 2 TiO_2_ (001) supercell was considered (186 atoms), with 38 water molecules. A 2 × 1 TiO_2_ (100) surface has been put in contact with the water box containing 45 water molecules and resulting in 219 atoms in the simulation cell. In each case, the starting configuration of the water box was containing seven water layers. The sampling of the reciprocal space in this case has been done to the gamma point due to the size of the simulation cells, after checking that this is an acceptable choice.^56^ We evolved two trajectories for each system. In the first one, the starting configuration is characterized by molecular adsorption of water, with intact water molecules adsorbed on surface Ti sites. In the second, we considered dissociated water molecules, where OH species are adsorbed on Ti sites, and H atoms are adsorbed on surface oxygen atoms.^8^

Also in the choice of the propagation time, one must find a compromise between accuracy and computational effort. The calculated acidic constants in eqs 4 and 5 are evaluated from quantities obtained from averages along the AIMD trajectory. The error has been evaluated by means of the blocking analysis as done in previous studies.^36,37,57^ Within our simulation time, the error is always of about 0.1 eV, which is of the same order of accuracy of the whole methodology.^8^ Also, previous studies showed that such a propagation time allows us to reproduce the experimental point of zero charge of several oxides, such as BiVO_4_, MgO, anatase TiO_2_, and γ-Al_2_O_3_.^8,9^ Because of the aforementioned reasons, the propagation time can be considered sufficient to extract reliable information on the surface chemistry. This evidence agrees with previous studies on solid/water interfaces based on AIMD trajectories, based on propagation times of about 4–8 ps.^51,55,58,59^ We must stress, however, that about 1 order of magnitude larger propagation times would be necessary for a full statistical sampling.^41^ Moreover, the fact that 5 ps can be considered sufficient for this specific purpose does not imply that this is enough to properly reproduce other aspects of surface chemistry. Seminal works on rutile (110)/water interfaces showed that the full treatment of the system in aqueous solution requires us to propagate trajectories of about 25 ps.^52,53^

**Figure 1:**
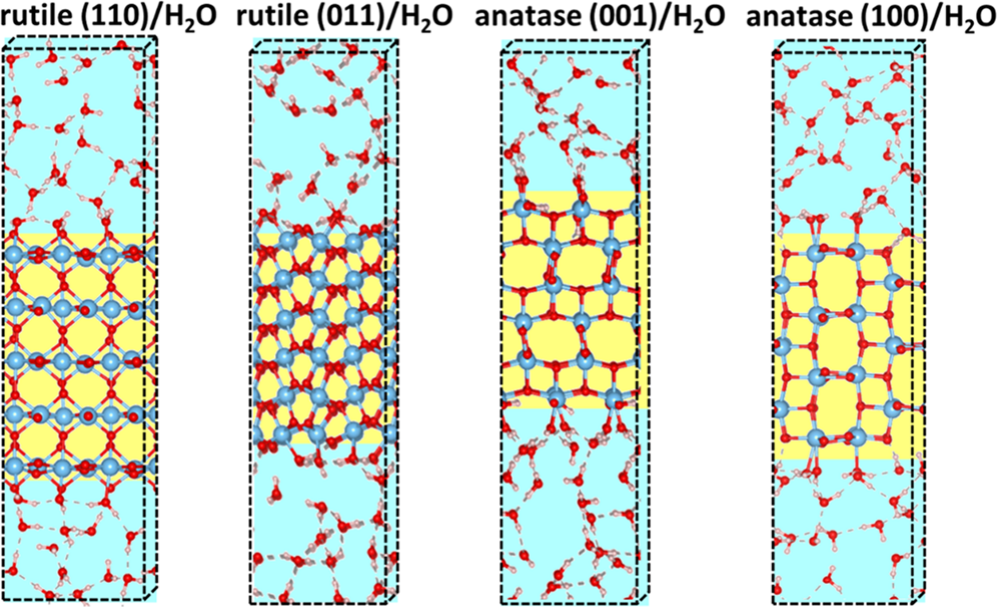
Snapshot of rutile (110)/water, rutile (011)/water, anatase (001)/water, and anatase (100)/water interfaces.

After a propagation time of 5 ps, the two trajectories of rutile (110), rutile (011), and anatase (001) lead to very similar dissociation fractions. The only exception is represented by anatase (100) for which we found that after 5 ps two different pictures are possible and the energies are almost comparable. In the first case, we found both adsorbed and dissociated water molecules at the surface, while in the second case, a fully dissociated water layer is present. Further studies are planned for this system to better characterize these features. In the present work, we decided to proceed with the propagation of charged trajectories from the first case since it contains both molecularly and dissociated water molecules. Figure 1 shows a snapshot of the rutile (110)/water, rutile (011)/water, anatase (001)/water, and anatase (100)/water interfaces.

The main working equations to determine acidic constants and point of zero charge are reported below. More details can be found in the Supporting Information (SI) and in refs (8) and.^9^ In this study, we adopt the formulation proposed by Pasquarello and co-workers, based on the grand canonical formulation of species in solution; see eqs S1–S3.^8,36^ In particular, one can propagate ab initio molecular dynamics AIMD trajectories of neutral TiO_2_/water interface, and the same by adding or removing an extra proton at the interface.^8^ This allows us to describe acid–base reactions described below. Each AIMD trajectory is propagated with a constant number of particles. A uniform charged background was added to compensate the extra charge. We neglected finite size effects, which are often very small (0.01–0.02 eV) for water and materials with a significant dielectric constant.^41^ It must be said that a change in the charge state of a system can imply a shift in the potential of electrodes, with possible consequences when studying electrocatalytic processes.^60^ On the surface of TiO_2_, a proton from water can be attached to a surface oxygen atom (O_s_H^+^), and a water molecule can adsorb on a titanium one (H_2_O_Ti_). The acid–base equilibria can then be written according to the following reactions
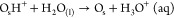

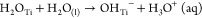


Described by the corresponding equilibrium constants
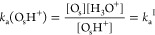
1
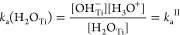
2

If we label the total number of exposed titanium and oxygen sites as *C*_Ti_ and *C*_O_, the pH corresponding to PZC (pH_PZC_) is equal to^8^
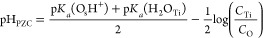
3

The derivation of the working equations for equilibrium constants is reported in eqs S4–S14. They can be calculated as
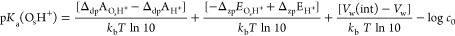
4
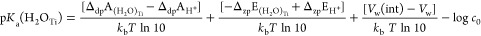
5

Each term of the equations is averaged along AIMD simulation of TiO_2_/water interfaces. The first term is the integral for the deprotonation of an adsorbed proton of the surface Δ_dp_A_O_s_H^+^_, of an adsorbed water molecule on a surface Δ_dp_A_(H_2_O)Ti_, with respect to the same property evaluated in acidic bulk water Δ_dp_A_H^+^_. These integrals are obtained by propagating AIMD simulations of a neutral TiO_2_/water interface, and the same having an extra proton and a hydroxyl ion adsorbed on the surface.^8,9^ The second term is related to the zero-point energy correction, and the third term is associated with the difference between the macroscopic averaged electrostatic potential on the water side of the neutral TiO_2_/water interface and the same of bulk water.^8,9^ c_0_ is the concentration of water molecules in water solution .

The same approach can be used to calculate the relative stability of molecular and dissociative adsorption of water molecules.^8,9^ This is derived in the SI, eqs S16 and S17. Indeed, we can estimate the energy (Δ*A*_d_) to dissociate a molecularly adsorbed water molecule as
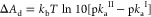
6

Such an approach is somewhat related to the very successful computational hydrogen electrode (CHE) method of Norskov and co-workers,^61,62^ where the dissociation free energy of water can be obtained from the calculation of minimum-energy structures followed by the inclusion of thermodynamic correction. The current methodology already accounts for the fluxional behavior of water, resulting in the presence of several local minima very close in energy. Once evaluated the acidic constants and the point of zero charge, it is possible to study the amount of charged species and free surface sites as a function of the pH. Full details and derivation are reported in the SI and in ref (9). Indeed, the molar fraction of the species on the surface is
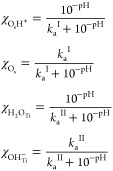
7

## 3 Results and Discussion

### 3.1 Neutral TiO_2_/H_2_O Interfaces

**Figure 2:**
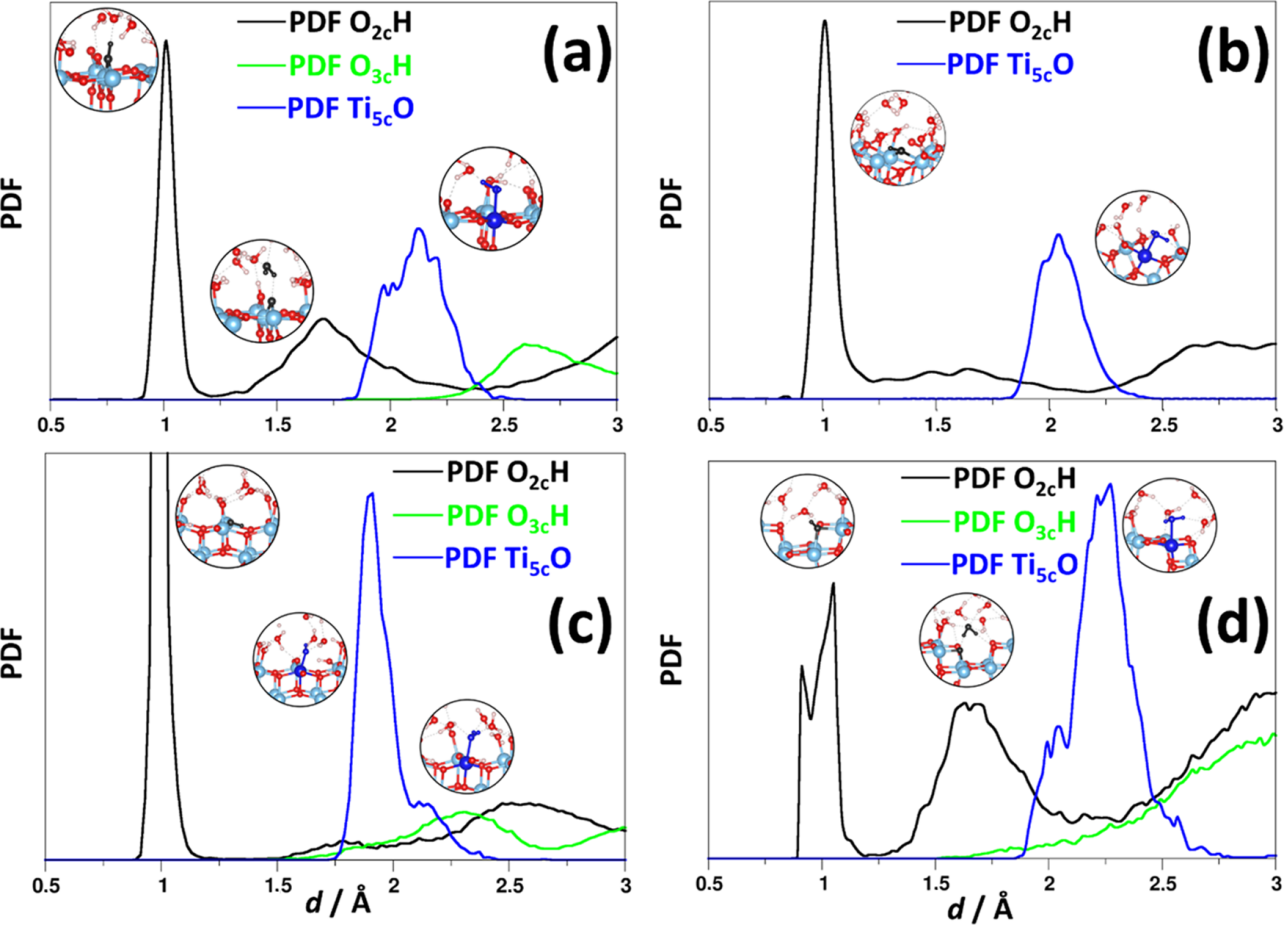
Calculated pair distribution function (PDF) between O_2c_ and H atoms (black solid line), O_3c_ and H atoms (green solid line), and surface Ti and O atoms of water (blue solid line) for TiO_2_/water interfaces. (a–d) Rutile (110), rutile (011), anatase (001), and anatase (100), respectively.

We start from the analysis of the rutile (110)/H_2_O interface. After 5 ps, the fraction of dissociated water molecules on the surface reaches a stable value of about 25%. Figure 2 shows the pair distribution function (PDF) between surface atoms and water. The peak at 0.99 Å characterizes the formation of cO_s_–H bonds, due to water chemisorption. The calculated coordination number is 0.25, in agreement with the fraction of water dissociated on the surface. The coordination number is evaluated by integrating the PDF.^63^ A second peak at 1.7 Å is due to hydrogen bonding between the O_2c_ atoms and water molecules. The calculated coordination number is 0.66; therefore, a significantly strong network of hydrogen bonding forms between rutile (110) and water. O_3c_ atoms on the surface are nearly inert, as evinced by the large distances shown in Figure 2a. The PDF between titanium atoms and oxygen species shows a peak at 2.1 Å due to the interaction with adsorbed H_2_O and OH^–^ species arising from water dissociation. The calculated coordination number is one; therefore, on average, each titanium atom is covered. The first layer of water molecules on the surface arranges in dimers, as already reported, with a typical O–O distance of 3.0 Å.^24^ The calculated free energy of water dissociation is 0.53 eV, indicating that the chemisorption process is endergonic. This number is comparable to that of anatase (101)/H_2_O interface, 0.48 eV.^9^

The rutile (011)/water interface displays large changes with respect to the (110)/water one. Indeed, after 5 ps, the dissociation fraction is significantly larger, about 50%, as also suggested by the calculated coordination number between O_2c_ and H. The corresponding PDF is reported in Figure 2b. Consistently, the calculated water dissociation energy is 0.21 eV, about half of that of rutile (110). Hydrogen bonding is present, as evinced by the broad signal at 1.7 Å, where each O_2c_ atom contributes to 0.5 hydrogen bonds. Similar to the (110) surface, each Ti_5c_ atom is on average interacting with a OH^–^ or a H_2_O species, with a Ti_5c_–O distance of 2.1 Å.

Moving to the anatase surfaces, the analysis of TiO_2_ (001)/water trajectory indicates that there is a larger tendency for water dissociation.^20,23^ Indeed, after 5 ps, the dissociation fraction is about 75%, due to the higher reactivity of this surface. Indeed, the calculated dissociation free energy is 0.32 eV, much lower than for rutile (110) and anatase (101). In this latter case, it has been shown that water tends to adsorb molecularly on the surface.^22^Figure 2c reports the calculated PDFs, where it is possible to identify the main O_2c_–H peak at about 0.99 Å, with an average coordination number of 0.75. The peak at 1.91 Å is associated with the interaction between Ti_5c_ atoms and OH^–^ ions arising from water dissociation, with the same coordination number, 0.75, confirming the calculated dissociation fraction. The small shoulder at 2.1 Å is attributed to the small number of water molecules on the surface, and indeed the average coordination number is 0.25. All titanium atoms on the surface are interacting on average with one water molecule. The peak at 1.7 Å is due to hydrogen bonding between O_2c_ and water, and the calculated coordination number is 0.2. Interestingly, while there is a larger dissociation of water molecules on the anatase (001) surface than on the rutile (110), α = 0.75 and α = 0.25, respectively, there is a consequent decrease of the available sites for H-bonding, which results in a much smaller coordination number, 0.2 and 0.66 for anatase (001) and rutile (110), respectively.

We now analyze the anatase (100)/water interface. In this case, the most likely interaction is physisorption, since the dissociation fraction is low, 12%, comparable to that of anatase (101),^9^ at variance to anatase (001). Indeed, the calculated dissociation energy is higher than that of anatase (001), Δ*A* = 0.78 eV. As already mentioned, a conclusive picture of water adsorption on solid surfaces would require much longer simulation times, but this aspect goes beyond the purpose of this study. In fact, further work is needed to fully elucidate the anatase (100)/water interface. Figure 2d reports the analysis of the PDF, where the typical O_2c_–H, Ti_5c_–OH, and Ti_5c_–H_2_O distances are nearly the same as of anatase (001). Interestingly, the amount of hydrogen bonding between O_2c_ atoms and water is quite high, about 0.5, which is comparable to that of rutile (110).

### 3.2 Acidity of TiO_2_ Surfaces

As mentioned above, the propagation of two trajectories, one including an extra proton attached to the surface, and the second that includes a hydroxyl species, allow us to evaluate the acidity of the different surface sites and the point of zero charge. In addition, starting from the calculated p*k*_a_’s, it is possible to derive the amounts of H^+^, OH^–^, and H_2_O species present on the surfaces. Table 2 reports the calculated equilibrium constants and points of zero charge. Tables S1 and S2 report the calculated deprotonation energies and zero-point energy corrections. For the most stable surfaces of rutile and anatase, the (110) and (101), respectively, we compute a pH_PZC_ of 5.39 and 5.95 (see Table 2), indicating a slightly acidic behavior.^64^ Both values are close to the reported experimental estimates of a pH_PZC_ about 6. Notice, however, that the fact that the pH_PZC_ of rutile is about 0.5 pH units lower than that of anatase surfaces finds support in some experimental observations.^65^ Of course, what is measured experimentally is an average of the behavior of the variously exposed facets in TiO_2_ nanoparticles, and this in turn depends on the preparation of the sample. With our calculations, on the contrary, we can provide a detailed analysis of the dependence of the pH_PZC_ on the exposed surface.

**Table 2:** Calculated Deprotonation Integrals with respect to Δ_dp_A_H^+^_, Equilibrium Constants (p*k*_a_^I^, p*k*_a_^II^), and pH_PZC_ Compared with Available Experimental Values for Rutile (110), Anatase (001), and Anatase (100) Surfaces^a^

system	Δ_dp_A_O_s_H^+^_/eV	Δ_dp_A_H_2_O_M__/eV	p*k*_a_^I^	p*k*_a_^II^	pH_PZC_	pH_PZC_^EXP7,^^64,66^
rutile (110)	–0.64	–0.17	1.45	9.02	5.39	∼6^b^
rutile (011)	–0.52	–0.33	2.60	5.65	4.28	
anatase (001)	–0.72	–0.40	3.40	8.05	5.88	
anatase (100)	–1.15	–0.32	1.24	12.50	7.02	
anatase (101)	–1.12	–0.62	2.33	9.27	5.95	∼6^b^

a Results for anatase (101) taken from ref (9).

b The experimental values are averaged over the various surfaces exposed. Here, it is associated with the most stable surface of the TiO_2_ polymorph.

The point of zero charge of rutile (011), 4.28, is significantly lower than that of the most stable (110) surface, 5.39, indicating a stronger acidity of this surface. Moving to the anatase (001)/water interface, the calculated point of zero charge is 5.88, very similar to that of rutile and in agreement with that measured for anatase crystals.^7,66^ Interestingly, the behavior of the other anatase surface, (100), is substantially different, as reported experimentally.^67^ Indeed, the calculated point of zero charge, 7.02, is considerably higher than that of the other surfaces considered.

The different acidic behavior of the surface models considered has a direct implication on the amount of species present on the surfaces, a relevant aspect for reactions such as water splitting.^68,69^

**Figure 3:**
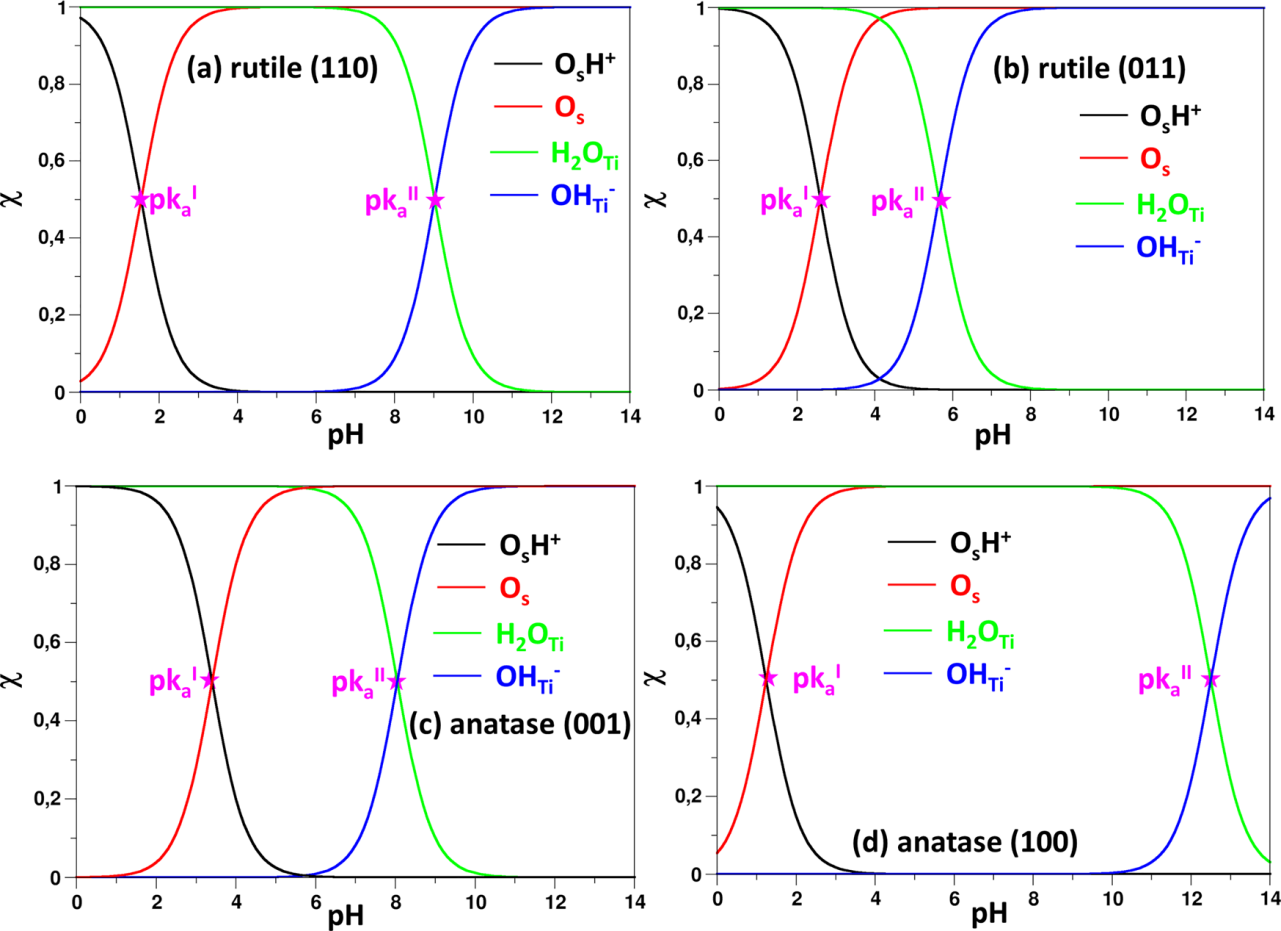
Speciation of adsorbed water (H_2_O), free surface oxygen sites (O_s_), and H^+^ and OH^–^ species as a function of pH on the surface of (a) rutile (110), (b) rutile (011), (c) anatase (001), and (d) anatase (100).

**Figure 4:**
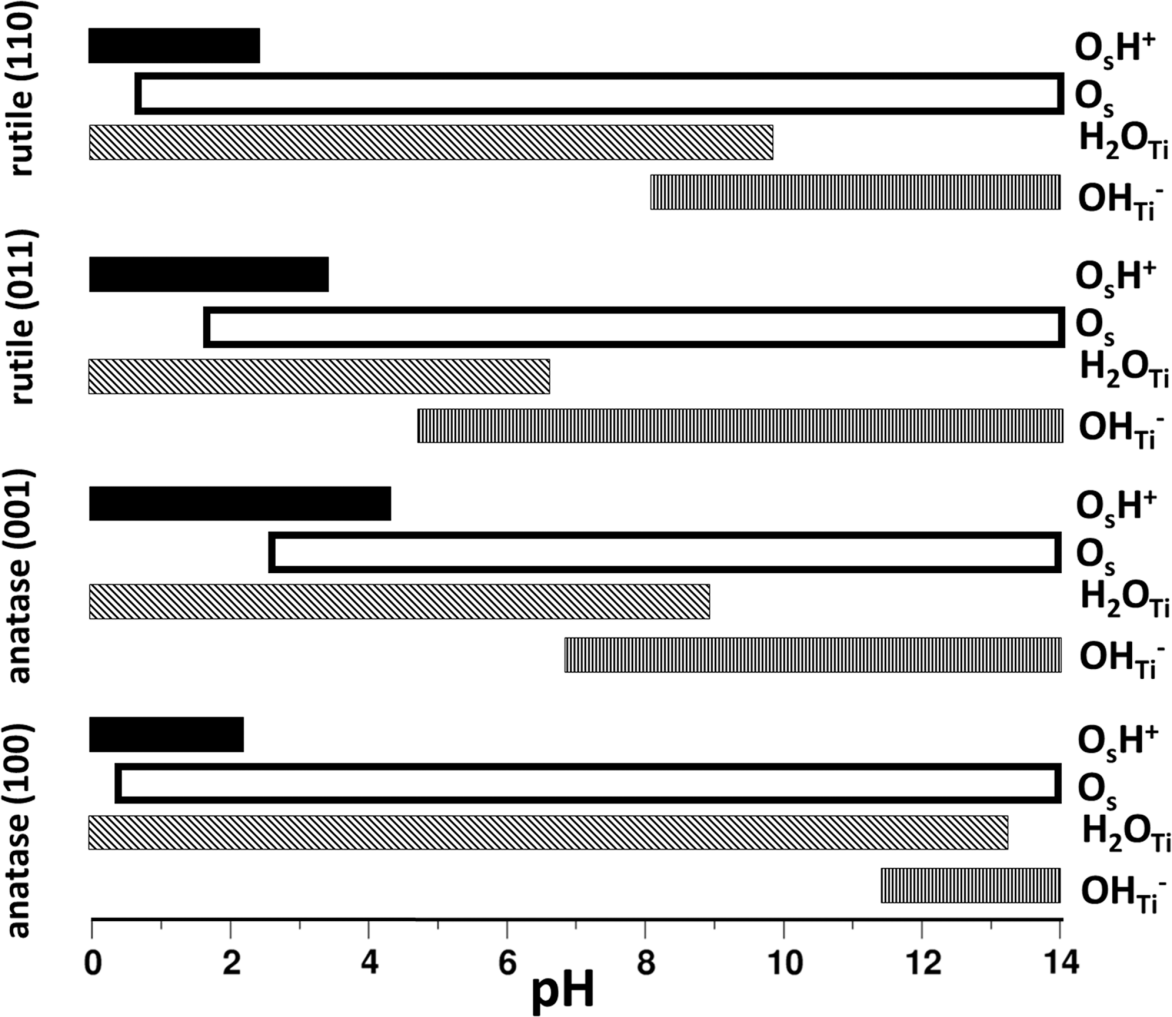
pH windows at which the H^+^, O_s_, H_2_O, and OH^–^ species are present on the surface. A threshold molar fraction of 10% has been adopted.

Figure 3 shows the trend of molar fractions of surface sites as a function of pH. Figure 4 depicts the pH window at which the species are available of the surface. A threshold value of 10% was adopted.^9^ The speciation diagrams are obtained from eqs S20 and S21 and are based on a post-process analysis depending on the calculated acidic constants.^9^ Therefore, the estimates should be taken with some care in analyzing absolute numbers, while they can provide some useful information when looking at trends and doing comparisons.

We see that for the rutile(110)/water interface, H^+^ ions are present on the surface only at a quite low pH, below 2.5, while at higher pH values, the surface oxygen atoms are expected to be free. At the same time, OH^–^ ions display larger stability since they are present on the surface at pH > 8. Given the large acidity of rutile (011), OH^–^ ions are found in a large pH window. Moving to the anatase (001)/water model, although the pH_PZC_ is quite similar to that of rutile (110), the different p*k*_a_’s imply a very different pH-dependent behavior. Water dissociation is more favorable on this surface, and indeed both H^+^ and OH^–^ species are likely to be present on the surface, for pH < 4.5 and pH > 7.0, respectively. The different surface chemistry is even more evident when comparing anatase (100) and (001) surfaces. H^+^ and OH^–^ species are unlikely to be present on the first surface, according to the energy cost to dissociate water molecules on the surface. H^+^ becomes abundant only at pH < 2.5, and OH^–^ for pH > 11.5. At the same time, the window where water molecules dominate is the largest among the four models since H_2_O is present on the surface for pH < 13. The pH-dependent behavior of the different facets can have implications for the catalytic activity. For instance, anatase crystals made by anatase/rutile junctions are found to be active for photocatalytic hydrogen production. This is mainly attributed to the improved charge carriers separation due to a favorable alignment of the band edges, where electrons are promoted to migrate toward the anatase (101) facet and holes toward the rutile (110) one.^70^ Our results indicate that the presence of H^+^ is more likely to occur on anatase (101) according to Table 2, which is an additional important requirement for high activity in hydrogen production.

## 4 Conclusions

In this work, we investigated the pH- and facet-dependent surface chemistry of TiO_2_ in aqueous environment by means of ab initio molecular dynamics simulations. We considered rutile (110), rutile (011), anatase (001), anatase (100), and anatase (101) surfaces. We first looked at the nature of the interaction of water and the surface, finding that the tendency toward chemisorption is strongly surface-sensitive. Water is preferentially adsorbed molecularly on rutile (110) and anatase (100), and it is more likely to dissociate on rutile (011) and anatase (001). The fraction of dissociated molecules correlates significantly with the computed dissociation energy on the surface. We used the grand canonical formulation of species in solution to estimate the acid–base equilibrium constants at the TiO_2_/water interface. This required the simulation of additional trajectories including explicitly an extra proton and a hydroxyl group in the supercell simulating the interface. The calculated constants were used to estimate the pH of point of zero charge, an important experimental quantity. Even in this case, the results indicate that the chemistry of water on titania is surface-sensitive. The pH_PZC_ of rutile surfaces is slightly lower than that of anatase, in line with experimental observations.^65^ The differences in equilibrium constants at the surface have relevant implications for the amount of H^+^, OH^–^, and H_2_O species that can be present on the surface as a function of pH. Further work will be dedicated to expanding the model to include more realistic 3D nanoparticles and extended defects such as steps and kinks.

## Supporting Information Available

Details and derivation of the methodology (Tables S1 and S2) (PDF)
