# Supplementary material for: pH- and Facet-Dependent Surface Chemistry of TiO2 in Aqueous Environment from First Principles
Source: ACS Appl Mater Interfaces. 2023 Feb 14;15(8):11216–24. doi: 10.1021/acsami.2c19273 (PMC9982820; doi:10.1021/acsami.2c19273)
Supplement: Supplementary file 1 — am2c19273_si_001.pdf [file am2c19273_si_001.pdf]

pH- and Facet-dependent Surface Chemistry of TiO_2 in Aqueous Environment from First Principles

Supporting Information

Farahnaz Maleki, Giovanni Di Liberto, and Gianfranco Pacchioni*

Dipartimento di Scienza dei Materiali, Università di Milano-Bicocca, via R. Cozzi 55, 20125 Milano, Italy

S1 Theoretical Framework

One is interested in the following acid-base equilibria when putting a material in contact with water:

$$O_s\text{-}H^+ + H_2O_{(l)} \rightarrow O_s + H_3O^+_{(aq)}$$

$$H_2O_{Ti} + H_2O_{(l)} \rightarrow OH_{Ti}^- + H_3O^+_{(aq)}$$

The first reaction corresponds to the acidity of exposed surface oxygen sites, and the second to the acidity of a water molecule adsorbed on the catalytic surface.

The two equilibrium constants are:

$$k_a(O_sH^+) = \frac{[O_s][H_3O^+]}{[O_sH^+]} = k_a{}^I \qquad \text{Eq. S1}$$

$$k_a(H_2O_{\mathrm{Ti}}) = \frac{[OH_{\mathrm{Ti}}^-][H_3O^+]}{[H_2O_{\mathrm{Ti}}]} = k_a{}^{II} \qquad \text{Eq. S2}$$

The point of zero charge (PZC), can be calculated from the equilibrium constants as:

$$pH_{PZC} = \frac{pk_a(O_sH^+) + pk_a(H_2O_{\mathrm{Ti}})}{2} - \frac{1}{2}log\left(\frac{C_M}{C_O}\right) \qquad \text{Eq. S3}$$

Where C_M and C_O are the total number of surface exposed Metal and Oxygen sites.

The equilibrium constants are obtained by means of atomistic simulations using the Grand Canonical Formulation for species in solution. The original derivation and application of the approach can be found in recent works by Pasquarello and co-workers.[1,2] The main step are also reported in the SI of the Ref. [3] and below.

If one in interested in a solid interfaced with water, it is possible to write the Gibbs formation energy of a species S in contact with water and adsorbed on the surface of the solid as:

$$G_f^q[S] = G^q[S] - G[ref] - \sum_i n_i\mu_i + q(\varepsilon_{v-w}(int) + \mu_e) \quad \text{Eq. S4}$$

Where $G^q[S]$ is the Gibbs free energy of the species S, $G[ref]$ is the Gibbs free energy of the reference interface system, n_i and μ_i are the number of atoms added/removed and their chemical potential, $\varepsilon_{v-w}(int)$ can be related to valence band edges of bulk liquid water by the following alignment relation $\varepsilon_{v-w}(int) = \varepsilon_{v-w} + V_w(int) - V_w$, where $V_w(int)$ and V_w are the macroscopic averages electrostatic potential of water in the interface and bulk systems respectively. μ_e is the chemical potential of the electron. In Eq. S4 we neglect finite size effects, that are often very small (0.01-0.02 eV) for water and for materials with a significant dielectric.[4,5] If we consider the Gibbs formation energy of an adsorbed proton on the surface (O_sH^+), we have:

$$G_f^q[O_sH^+] = G^q[O_sH^+] - G[ref] - \mu_H + (\varepsilon_{v-w} + +V_w(int) - V_w + \mu_e) \quad \text{Eq. S5}$$

The free energy difference $G^q[O_sH^+] - G[ref]$ can be obtained through the thermodynamic integration method as:

$$G^q[O_sH^+] - G[ref] = -\int_0^1 \langle \Delta_{dp}E_{O_sH^+}\rangle_\tau d\tau + \Delta_{zp}E_{O_sH^+} \quad \text{Eq. S6}$$

$$= -\Delta_{dp}A_{O_sH^+} + \Delta_{zp}E_{O_sH^+}$$

The first term is the integral associated to the deprotonation of an adsorbed H^+ on the surface, and the second term is the zero-point energy term accounting for the vibrational motion of adsorbed H^+. We can then explicit the electron chemical potential:

$$\mu_e = G_f^q[O_sH^+] + \Delta_{dp}A_{O_sH^+} - \Delta_{zp}E_{O_sH^+} + \mu_H - (\varepsilon_{v-w} + +V_w(int) - V_w) \qquad \text{Eq. S7}$$

At the same time, we express the electron chemical potential in an acid water solution as:

$$G_f\ [H^+] = k_bTln(c_0) + k_bTln10pH \qquad \text{Eq. S8}$$

Where c$_0$ is concentration of water molecules in water solution $c_0 = \frac{1000g}{18^g/_{mol}}$. If we rewrite the formation energy we have:

$$G_f\ [H^+] = G^q[H^+] - G[H_2O_{bulk}] - \mu_H + (\varepsilon_{v-w} + \mu_e) \qquad \text{Eq. S9}$$

$$= -\Delta_{dp}A_{H^+} + \Delta_{zp}E_{H^+} - \mu_H + (\varepsilon_{v-w} + \mu_e)$$

and therefore

$$-\Delta_{dp}A_{H^+} + \Delta_{zp}E_{H^+} - \mu_H + (\varepsilon_{v-w} + \mu_e) = k_bTln(c_0) + k_bTln10pH \qquad \text{Eq. S10}$$

We can write the electron chemical potential as:

$$\mu_e = \Delta_{dp}A_{H^+} - \Delta_{zp}E_{H^+} + \mu_H - \varepsilon_{v-w} + k_bTlnc_0 + k_bTln10pH \qquad \text{Eq. S11}$$

leading to:

$$\mu_e = \Delta_{dp}A_{H^+} - \Delta_{zp}E_{H^+} + \mu_H - \varepsilon_{v-w} + k_bTlnc_0 + k_bTln10pH \qquad \text{Eq. S12}$$

$$= k_bTln(\frac{c_{O_sH^+}}{c_{O_s}}) + \Delta_{dp}A_{O_sH^+} - \Delta_{zp}E_{O_sH^+} + \mu_H$$

$$- (\varepsilon_{v-w} + +V_w(int) - V_w)$$

$$0 = \Delta_{dp}A_{O_sH^+} - \Delta_{dp}A_{H^+} + \left(-\Delta_{zp}E_{O_sH^+} + \Delta_{zp}E_{H^+}\right) + V_w(int) - V_w - k_bTlnc_0 + \qquad \text{Eq. S13}$$

$$k_bTln10log\left(\frac{c_{H^+}c_{O_s}}{c_{O_sH^+}}\right).$$

We can rearrange the equation to obtain:

$$pk_a(O_sH^+) = \frac{[\Delta_{dp}A_{O_sH^+} - \Delta_{dp}A_{H^+}]}{k_bTln10} + \frac{[-\Delta_{zp}E_{O_sH^+} + \Delta_{zp}E_{H^+}]}{k_bTln10} + \frac{[V_w(int) - V_w]}{k_b\,Tln10} - logc_0. \qquad \text{Eq. S14}$$

The first term is associated to the deprotonation integrals of an adsorbed proton on the surface and of H^+ in water. The second term accounts for the zero-point correction. The third term accounts for the macroscopic averaged electrostatic potentials, and the last term corresponds to -1.74.

Equivalently, the equilibrium constant for an adsorbed water molecule on the surface is:

$$pk_a(H_2O_{Ti}) = \frac{[\Delta_{dp}A_{MH_2O} - \Delta_{dp}A_{H^+}]}{k_bTln10} + \frac{[-\Delta_{zp}E_{MH_2O} + \Delta_{zp}E_{H^+}]}{k_bTln10} + \frac{[V_w(int) - V_w - V_w(vac) + V_w]}{k_b\,Tln10} - logc_0. \qquad \text{Eq. S15}$$

If we want to evaluate the pKa of a proton in water solution, $pKa(O_sH^+)$, the equation simply becomes $pk_a(H^+) = -logc_0 = -1.74$ which is the well-known value for water. The third term can be obtained by propagating a trajectory of a neutral solid/water interface and extrapolating the corresponding averaged potential. The first two terms instead require the propagation of two additional trajectories, one with an extra proton adsorbed on the surface (O_sH^+), one with an adsorbed hydroxyl group (OH^-) on the catalytic surface.

The same approach can be used to calculate the relative stability of molecular and dissociative adsorption of water molecules. Indeed, we can estimate the energy to dissociate a water molecule molecularly adsorbed, according to the reaction:

$$H_2O_{Ti} + O_s \rightarrow OH_{Ti}^- + O_sH^+$$

The equilibrium constant:

$$k_d = \frac{[OH_{\mathrm{Ti}}^-][O_sH^+]}{[H_2O_{\mathrm{Ti}}][O_s]} = \frac{[OH_{\mathrm{Ti}}^-][O_sH^+]}{[H_2O_{\mathrm{Ti}}][O_s]}\frac{[H_3O^+]}{[H_3O^+]} = \frac{k_a{}^{II}}{k_a{}^{I}} \qquad \text{Eq. S16}$$

The Free energy describing the reaction is:

$$k_d = \frac{k_a{}^{II}}{k_a{}^{I}} = \frac{[OH_{\mathrm{Ti}}^-][O_sH^+]}{[H_2O_{\mathrm{Ti}}][O_s]} = e^{-\frac{\left(G_f\,[OH_{\mathrm{Ti}}^-] + G_f\,[O_sH^+] - G_f\,[H_2O_{\mathrm{Ti}}] - G_f\,[O_s]\right)}{k_bT}} = e^{-\frac{\Delta A_d}{k_bT}} \qquad \text{Eq. S17}$$

Combining the last two Eqs. we obtain:

$$\Delta A_d = k_b T ln10 [pk_a{}^{II} - pk_a{}^{I}] \quad \text{Eq. S18}$$

A positive value of ΔA_d indicates that the molecular adsorption of water is more favourable that the dissociative one, and a negative value implies the opposite.

The deprotonation integrals are complex to evaluate and need to run constrained molecular dynamics simulations. Here, we adopt a linear approximation that has been shown to produce negligible errors in the deprotonation integrals.[2,6]

$$\Delta_{dp} A_{O_sH^+} = \int_0^1 \langle \Delta_{dp} E_{O_sH^+} \rangle_\tau d\tau = \frac{\langle \Delta_{dp} E_{O_sH^+} \rangle_0 + \langle \Delta_{dp} E_{O_sH^+} \rangle_1}{2} \quad \text{Eq. S19}$$

$$\Delta_{dp} A_{H_2O_{\mathrm{Ti}}} = \int_0^1 \langle \Delta_{dp} E_{H_2O_{\mathrm{Ti}}} \rangle_\tau d\tau = \frac{\langle \Delta_{dp} E_{H_2O_{\mathrm{Ti}}} \rangle_0 + \langle \Delta_{dp} E_{H_2O_{\mathrm{Ti}}} \rangle_1}{2}$$

$\langle \Delta_{dp} E_{O_sH^+} \rangle_0$ and $\langle \Delta_{dp} E_{H_2O_{\mathrm{Ti}}} \rangle_0$ are the vertical deprotonation energies of an adsorbed proton and of an adsorbed water molecule on the surface, respectively. They can be evaluated by sampling along the trajectory a certain number of configurations. In our case we sample and average 1000 configurations. The first term, $\langle \Delta_{dp} E_{O_sH^+} \rangle_0$ is calculated by detaching the extra proton on the surface in the protonated trajectory, *i.e.* by removing it and recalculating the total energy of the system by keeping the nuclei frozen. The second term, $\langle \Delta_{dp} E_{H_2O_{\mathrm{Ti}}} \rangle_0$ is calculated similarly, *i.e.* by removing a proton from an adsorbed water molecule in the neutral solid/water interface and recalculating the energy of the system at each configuration sampled. The remaining two terms $\langle \Delta_{dp} E_{O_sH^+} \rangle_1$ and $\langle \Delta_{dp} E_{H_2O_{\mathrm{Ti}}} \rangle_1$ are the energy for proton addition, and it would require the simulation of constrained molecular dynamics.[2,7] Here, we adopt the strategy proposed by Pasquarello and co-workers that allows to circumvent the problem, without any relevant loss of accuracy (the error is of the order of 0.05 eV).[1] The basic idea to evaluate $\langle \Delta_{dp} E_{O_sH^+} \rangle_1$ is to sample a sufficiently large number of configurations of the neutral solid/water interface and add an extra proton close to the surface site, O_s, and relax the extra proton at fixed nuclei positions. In this work, 1000 configurations are sampled. Similarly, $\langle \Delta_{dp} E_{H_2O_{\mathrm{Ti}}} \rangle_1$ is calculated by adding an extra proton close to an adsorbed hydroxyl group

on the surface, OH_{Ti}^-, and relax the proton at fixed nuclei positions. The zero-point energy correction $\Delta_{zp}E_{O_sH^+}$ is accounted for by performing vibrational frequency calculations in a harmonic fashion, according to the reaction $O_s\text{-}H^+ + H_2O_{(l)} \rightarrow O_s + H_3O^+_{(aq)}$. As for the previous quantities many configurations (1000) are sampled along the trajectories. Similarly, $\Delta_{zp}E_{H_2O_{Ti}}$ is calculated according to the reaction $H_2O_{Ti} + H_2O_{(l)} \rightarrow OH_{Ti}^- + H_3O^+_{(aq)}$.

The speciation diagrams reported in Figure 3 can be obtained as follows.[1,3] We can write the total concentration of surface oxygen and titanium atoms as:

$$c_{Os} = c_{O_s} + c_{O_sH^+} \qquad \text{Eq. S20}$$

$$c_{Ti} = c_{H2O_{Ti}} + c_{OH^-{}_{Ti}}$$

where c_{O_s} and $c_{O_sH^+}$ indicate the O_s atoms either free or bound to H^+, and $c_{OH^-{}_M}$ and c_{H2O_M} the metal sites bound to a hydroxyl group or to a water molecule. If we combine them with the expression of the acidic constants:

$$k_a(O_sH^+) = \frac{[O_s][H_3O^+]}{[O_sH^+]} = k_a{}^I$$

$$k_a(H_2O_{Ti}) = \frac{[OH_M^-][H_3O^+]}{[H_2O_{Ti}]} = k_a{}^{II}$$

one can correlate the concentration of the species at the surface with the pH:

$$c_{O_sH^+} = c_O \frac{10^{-pH}}{k_a{}^I + 10^{-pH}} \qquad \text{Eq. S21}$$

$$c_{O_s} = c_O \frac{k_a{}^I}{k_a{}^I + 10^{-pH}}$$

$$c_{H_2O_M} = c_M \frac{10^{-pH}}{k_a{}^{II} + 10^{-pH}}$$

$$c_{OH^-{}_M} = c_M \frac{k_a{}^{II}}{k_a{}^{II} + 10^{-pH}}$$

Eventually, it is possible to use molar fraction in place of concentrations, Eq S22 and Eq. 7.

$$\chi_{O_sH^+} = \frac{10^{-pH}}{k_a{}^I + 10^{-pH}} \qquad \text{Eq. S22}$$

$$\chi_{O_s} = \frac{k_a{}^I}{k_a{}^I + 10^{-pH}}$$

$$\chi_{H_2O_{Ti}} = \frac{10^{-pH}}{k_a{}^{II} + 10^{-pH}}$$

$$\chi_{OH^-{}_{Ti}} = \frac{k_a{}^{II}}{k_a{}^{II} + 10^{-pH}}$$

S2 Calculated point of zero charge

Table S1: Calculated properties of Rutile (110)/H_2O, Rutile (011)/H_2O, anatase TiO_2 (001)/H_2O, anatase TiO_2 (100)/H_2O, and anatase TiO_2 (101)/H_2O interfaces. From the left to the right are reported ΔV_w referred to the vacuum reference, deprotonation integrals $\Delta_{dp}A_{O_sH^+}$, $\Delta_{dp}A_{O_sH^+}$with respect to $\Delta_{dp}A_{O_sH^+}$ and $\Delta_{dp}A_{O_sH^+}$ respectively, Zero-point energy correction terms $\Delta_{ZPE}E_{O_sH^+}$ and $\Delta_{ZPE}E_{H_2O_M}$, and c_0.

System	ΔV_w / eV	$\Delta_{dp}A_{O_sH^+}$ / eV	$\Delta_{dp}A_{H_2O_M}$ / eV	$\Delta_{ZPE}E_{O_sH^+}$ / eV	$\Delta_{ZPE}E_{H_2O_M}$ / eV	Log(c_0)
R (110)	0.88	-0.64	-0.17	-0.02	0.04	-1.74
R (011)	0.83	-0.52	-0.33	-0.01	-0.01	-1.74
A (001)	1.10	-0.72	-0.40	-0.02	-0.02	-1.74
A (100)	1.33	-1.15	-0.32	0.03	-0.02	-1.74
A (101)	1.43	-1.12	-0.62	-0.03	-0.05	-1.74

Table S2: Calculated equilibrium constant of Rutile (110)/H_2O, Rutile (011)/H_2O, anatase TiO_2 (001)/H_2O, anatase TiO_2 (100)/H_2O, and anatase TiO_2 (101)/H_2O interfaces. From the left to the right are reported c_o/c_M, pk_a^I, pk_a^{II}, and pH_{PZC}.

System	c_o/c_M	pk_a^I	pk_a^{II}	pH_{PZC}
R (110)	2	1.45	9.02	5.39
R (011)	2	2.60	5.65	4.28
A (001)	2	3.40	8.05	5.88
A (100)	2	1.24	12.50	7.02
A (101)	2	2.33	9.27	5.95

References

(1) Ambrosio, F.; Wiktor, J.; Pasquarello, A. PH-Dependent Surface Chemistry from First Principles: Application to the BiVO 4 (010)–Water Interface. *ACS Appl. Mater. Interfaces* **2018**, *10* (12), 10011–10021. https://doi.org/10.1021/acsami.7b16545.

(2) Ambrosio, F.; Miceli, G.; Pasquarello, A. Redox Levels in Aqueous Solution: Effect of van Der Waals Interactions and Hybrid Functionals. *J. Chem. Phys.* **2015**, *143* (24), 244508. https://doi.org/10.1063/1.4938189.

(3) Di Liberto, G.; Maleki, F.; Pacchioni, G. PH Dependence of MgO, TiO 2 , and γ-Al 2 O 3 Surface Chemistry from First Principles. *J. Phys. Chem. C* **2022**, *126* (24), 10216–10223. https://doi.org/10.1021/acs.jpcc.2c02289.

(4) Guo, Z.; Ambrosio, F.; Pasquarello, A. Evaluation of Photocatalysts for Water Splitting through Combined Analysis of Surface Coverage and Energy-Level Alignment. *ACS Catal.* **2020**, *10* (22), 13186–13195. https://doi.org/10.1021/acscatal.0c03006.

(5) Guo, Z.; Ambrosio, F.; Chen, W.; Gono, P.; Pasquarello, A. Alignment of Redox Levels at Semiconductor–Water Interfaces. *Chem. Mater.* **2018**, *30* (1), 94–111. https://doi.org/10.1021/acs.chemmater.7b02619.

(6) Ambrosio, F.; Miceli, G.; Pasquarello, A. Structural, Dynamical, and Electronic Properties of Liquid Water: A Hybrid Functional Study. *J. Phys. Chem. B* **2016**, *120* (30), 7456–7470. https://doi.org/10.1021/acs.jpcb.6b03876.

(7) Cheng, J.; Sprik, M. Alignment of Electronic Energy Levels at Electrochemical Interfaces. *Phys. Chem. Chem. Phys.* **2012**, *14* (32), 11245. https://doi.org/10.1039/c2cp41652b.
